# Vitamin D Status Determines Cardiometabolic Effects of Testosterone Replacement Therapy in Men with Late-Onset Hypogonadism

**DOI:** 10.3390/nu17061013

**Published:** 2025-03-13

**Authors:** Robert Krysiak, Karolina Kowalcze, Witold Szkróbka, Bogusław Okopień

**Affiliations:** 1 Department of Internal Medicine and Clinical Pharmacology, Medical University of Silesia, Medyków 18, 40-752 Katowice, Poland; wszkrobka@sum.edu.pl (W.S.); bokopien@sum.edu.pl (B.O.); 2 Department of Pediatrics in Bytom, Faculty of Health Sciences in Katowice, Medical University of Silesia, Stefana Batorego 15, 41-902 Bytom, Poland; kkowalcze@sum.edu.pl; 3 Department of Pathophysiology, Faculty of Medicine, Academy of Silesia, Rolna 43, 40-555 Katowice, Poland

**Keywords:** cardiometabolic risk, late-onset hypogonadism, testosterone replacement, vitamin D

## Abstract

***Background/Objectives*:** Low testosterone levels and low vitamin D status are associated with increased cardiometabolic risk. The purpose of this study was to investigate whether vitamin D status determines the cardiometabolic effects of testosterone replacement therapy. ***Methods*:** The study population consisted of three groups of men with late-onset hypogonadism: vitamin D-naive individuals with 25-hydroxyvitamin D levels between 20 and 30 ng/mL (group I), males with 25-hydroxyvitamin D levels between 30 and 60 ng/mL receiving vitamin D supplementation because of previous low vitamin D status (group II), and vitamin D-naïve subjects with 25-hydroxyvitamin D levels between 30 and 60 ng/mL (group III). Circulating levels of total testosterone, 25-hydroxyvitamin D, glucose, insulin, lipids, uric acid, high-sensitivity C-reactive protein (hsCRP), homocysteine, fibrinogen, and urinary albumin-to-creatinine ratio (UACR) were assessed before and six months after intramuscular testosterone administration (250 mg every three weeks). ***Results*:** Group I differed from the remaining groups in baseline values of 25-hydroxyvitamin D, hsCRP, homocysteine, fibrinogen, UACR, and the Framingham Risk Score. In all three groups, testosterone injections increased plasma testosterone levels and had a neutral effect on 25-hydroxyvitamin D concentration. In groups II and III, the drug improved insulin sensitivity and reduced LDL cholesterol, uric acid, hsCRP, homocysteine, fibrinogen, and UACR. In group I, the impact of testosterone was limited to a small decrease in HDL cholesterol and hsCRP. Only in groups II and III did testosterone reduce the Framingham Risk Score. There were no differences in the strength of testosterone action between both groups. In groups II and III, the replacement-induced changes in insulin sensitivity, LDL cholesterol, uric acid, hsCRP, homocysteine, fibrinogen, UACR, and the Framingham Risk Score positively correlated with 25-hydroxyvitamin D concentration. ***Conclusions*:** The study results suggest that the cardiometabolic effects of exogenous testosterone in men with testosterone deficiency may be determined by vitamin D status.

## 1. Introduction

Human male aging is associated with a progressive decrease in testosterone production. In men above 40 years of age, the rate of decline in total and bioavailable testosterone levels is estimated at, respectively, 0.8 and 2% per year [1]. In some middle-aged and elderly men, low testosterone levels are accompanied by a ray of clinical symptoms, including low sexual desire, erectile dysfunction, poor morning erection, depression, hot flashes, inability to perform vigorous activity, cognitive disturbances, fatigue, and decreased vital energy [2,3]. Low testosterone levels in aging men are also associated with a decrease in muscle mass and strength, increased adiposity, decreased insulin sensitivity, osteopenia or osteoporosis, and impaired quality of life. The combination of subnormal testosterone concentration and different sexual, physical, and psychological symptoms is known under the name of late-onset hypogonadism (LOH), although there are also alternative names (androgen deficiency in the aging male, partial androgen deficiency in the aging male, or andropause) [4,5]. The prevalence of LOH in men aged 40–70 years varies from 30% to 40% and is highest in developed countries, although many cases are overlooked or misdiagnosed [5]. Low testosterone levels are associated with increased risk of low-grade systemic inflammation, enhanced coagulation, flow-mediated dilation of the brachial artery, and greater intima-media thickness [6,7]. In some studies, low testosterone levels correlated with increased risk of cardiovascular mortality, cardiovascular disease, metabolic syndrome, and type 2 diabetes [8,9,10]. These cardiometabolic effects of testosterone are explained by the interplay between genomic and non-genomic mechanisms of testosterone action that may partially overlap [11]. The former are associated with the passage of testosterone through the cellular membrane and binding to the androgen receptor, leading to activation or repression of specific genes [12]. In turn, the latter mechanisms, particularly important for vasodilatation, are independent of the ligand-dependent transactivation function of nuclear receptors, have a more rapid action onset, and seem to be related to the interaction with membrane proteins, receptors, and/or ion channels [11]. Despite the unfavorable impact of testosterone deficiency on men’s health, cardiometabolic effects of testosterone replacement are not so clear-cut. In some prospective cohort studies, testosterone substitution was associated with a significant reduction in all-cause mortality, myocardial infarction, and stroke [13,14,15]. However, other clinical studies showed either its neutral effect [16] or even an increased risk of adverse cardiovascular outcomes [17]. Similarly, depending on the study, testosterone replacement therapy improved insulin sensitivity and glycemic control in men with low testosterone levels [18] or did not affect insulin sensitivity [19]. These differences in obtained results suggest that genetic or environmental factors may modulate the cardiometabolic effects of testosterone replacement therapy.

One of the potential factors determining the strength of testosterone action is vitamin D (calciferol). Cardiomyocytes, endothelial cells, and smooth muscle cells express 1α-hydroxylase, a key enzyme in vitamin D activation, and high-affinity receptors for calcitriol, an active vitamin D metabolite [20], suggesting that disturbances in calciferol homeostasis may play an important role in the pathogenesis of cardiovascular disorders. High concentrations of vitamin D were associated with a 43% reduction in the prevalence of all cardiometabolic diseases, as well as with reduced prevalence of cardiovascular disease, type 2 diabetes, and metabolic syndrome [21]. An analysis of the Third National Health and Nutrition Examination Survey (NHANES III) showed that the highest quartile compared to the lowest quartile of 25-hydroxyvitamin D levels was inversely associated with coronary artery disease and all-cause mortality [22]. Individuals with congestive heart failure had markedly reduced levels of both 25-hydroxyvitamin D and calcitriol [23]. Reduced levels of 25-hydroxyvitamin D were linked to an elevated likelihood of developing ischemic stroke and with an unfavorable prognosis in patients who developed stroke [24]. Observational studies showed a significant inverse association between 25-hydroxyvitamin D and the incidence of type 2 diabetes [25]. Lastly, vitamin D deficiency was found to be a risk factor for endothelial dysfunction [26] and was often associated with ultrasonographic features of subclinical atherosclerosis [27]. Increased values of blood pressure (both systolic and diastolic), decreased concentrations of HDL cholesterol, and increased values of glucose, insulin, HOMA1-IR, and triglycerides in individuals with vitamin D deficiency have been reported already in adolescence [28].

The effect of vitamin D on the hypothalamic-pituitary-testicular axis has not been unequivocally established. Positive associations between 25-hydroxyvitamin D and testosterone concentrations and inverse associations between 25-hydroxyvitamin D and LH levels were reported in middle-aged or elderly men [29,30], but they were absent in young men [31]. In interventional clinical studies, there were no differences in testosterone levels between individuals randomized to vitamin D and randomized to placebo [32,33]. However, infertile males with 25-hydroxyvitamin D levels not exceeding 50 nmol/L were characterized by a higher testosterone/LH ratio after treatment with vitamin D and calcium than after placebo [34]. Lastly, calcitriol added to testicular cell cultures increased testosterone production [35], but the effect of chorionic gonadotropin on steroid hormone production in Leydig cells was unaffected by vitamin D co-treatment [36].

Concomitant testosterone deficiency and low vitamin D status may theoretically increase cardiometabolic risk to a greater extent than each of these conditions alone. To the best of our knowledge, no previous study has investigated the relationship between cardiovascular and metabolic effects of testosterone and vitamin D. However, previously we have reported that 25-hydroxyvitamin D levels were lower in patients with LOH not receiving exogenous testosterone [37], as well as that testosterone preparation potentiated the impact of vitamin D on thyroid antibody titers and the thyroid’s secretory capacity in men with testosterone deficiency [38]. Thus, we considered it interesting to investigate whether vitamin D status determines the cardiometabolic effects of testosterone substitution in men with testosterone deficiency, and answering this question was the aim of our study.

## 2. Materials and Methods

The study protocol was approved by the appropriate institutional review board. All procedures were conducted in agreement with the 1964 Helsinki Declaration and its following amendments. Written informed consent was provided by each participant after receiving a verbal and written description of the protocol. Because of its nature (the same drug in all patients independently of group assignment), the study did not have to be registered in the clinical trial registry.

### 2.1. Patients

The participants of our prospective matched cohort study were recruited among men aged 40 to 75 years old, with late-onset hypogonadism (LOH), defined as total testosterone concentrations less than 10.4 nmol/L (3.0 ng/mL) on two different occasions (at least six weeks apart) that coexisted with the following sexual manifestations: decreased frequency of morning erection, decreased frequency of sexual thoughts, and erectile dysfunction. All potential participants were required to comply for at least three months with the lifestyle modification.

**Figure 1:**
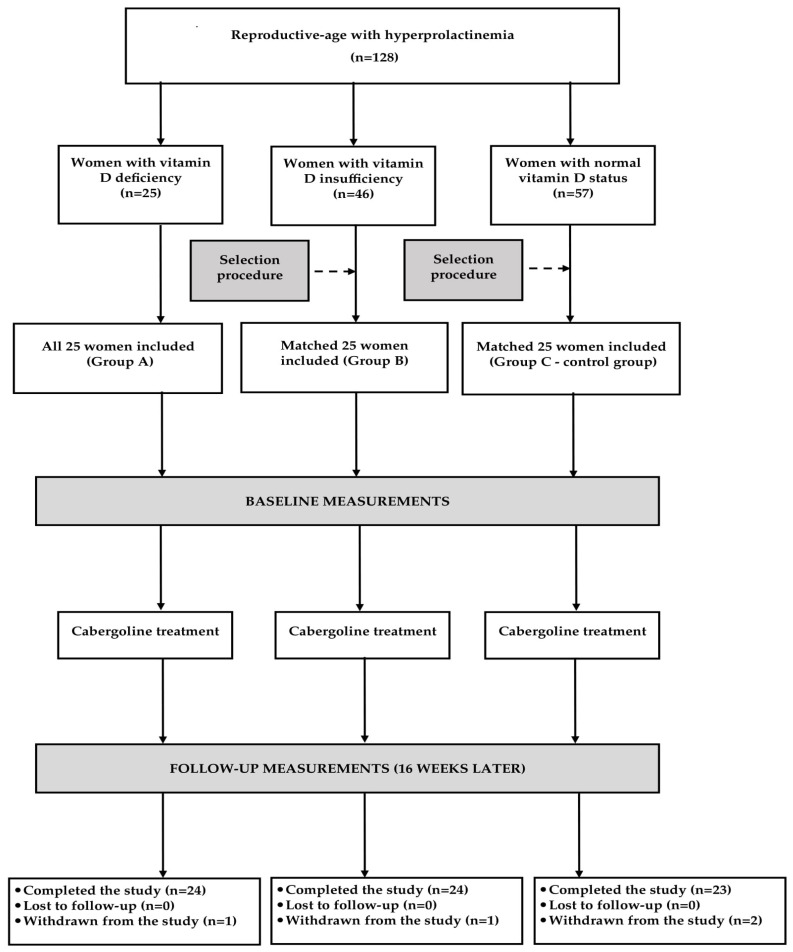
Flow of patients through the study.

Depending on plasma 25-hydroxyvitamin D levels, the participants were assigned to one of three study groups. Group I included vitamin D-naive individuals with vitamin D insufficiency (25-hydroxyvitamin D levels between 20 and 30 ng/mL). Group II consisted of males with normal calciferol status (25-hydroxyvitamin D levels between 30 and 60 ng/mL) receiving vitamin D supplementation (50–100 μg daily) because of previous vitamin D deficiency/insufficiency. Group III (the reference group) included vitamin D-naïve men with 25-hydroxyvitamin D levels between 30 and 60 ng/mL. For ethical reasons, vitamin D-naïve subjects with 25-hydroxyvitamin D levels below 20 ng/mL were not considered for enrollment. Prospective sample size calculation showed that at least 20 individuals per group were needed in order to detect a 20% between-group difference in all measured risk factors with 80% power at the 5% level of significance. The number of participants in each group was increased to 24 (by 20%) in order to account for possible losses and withdrawals. All vitamin D-naive individuals with vitamin D insufficiency meeting the remaining inclusion and exclusion criteria participated in the study. In turn, only some men with normal vitamin D status were recruited in order to match the study groups for age, body mass index (BMI), and baseline testosterone concentrations (Figure 1). The participating men were selected using an algorithm based on the minimum Euclidean distance rule. To minimize the effect of seasonal variation in levels of 25-hydroxyvitamin D and maybe also in levels of other outcome measures, similar numbers of men were recruited between January and March (6 in groups I and III and 7 in group II), between April and June (6 in each group), between July and August (7 in group I, 5 in groups II and III), and between September and December (5 in group I, 6 in group II, and 7 in group III).

Patients were ineligible for enrollment if they met any of the following criteria: prostate cancer, severe lower urinary tract symptoms (the American Urological Association International Prostate Symptom Score exceeding 19), baseline prostate-specific antigen greater than 4 ng/mL (or above 3 ng/mL in men at high risk of prostate cancer), breast cancer, myocardial infarction, acute coronary event, unstable angina, stroke or coronary revascularization procedure within 6 months preceding the study, severe heart failure (classes II–IV according to the New York Heart Association Functional Classification), uncontrolled arterial hypertension, hematocrit exceeding 50%, or untreated obstructive sleep apnea syndrome. We also excluded patients with diabetes, other endocrine disorders, renal or liver insufficiency, chronic inflammatory or autoimmune disorders, patients taking drugs known to interact with vitamin D or testosterone or known to affect cardiovascular risk, as well as subjects with poor compliance.

### 2.2. Study Design

All participants received testosterone replacement therapy and continued to follow the lifestyle modification. They were treated with 250 mg of a mixture of testosterone esters (30 mg of testosterone propionate, 60 mg of testosterone phenylpropionate, 60 mg of testosterone isocaproate, and 100 mg of testosterone caproate), which was administered intramuscularly every 21 ± 3 days for six months. Moreover, all patients assigned to group II received exogenous vitamin D preparations at the same dose as before commencement. Adherence to calciferol supplementation was checked every six weeks by pill counts of returned vitamin D tablets. Consumption of vitamin D with food was calculated based on the analysis of dietary diaries completed during the study. Other medications were allowed only if they were used for a maximum of seven days and not during the last six weeks of the study.

### 2.3. Laboratory Assays

All measurements were performed at baseline and again six months later (Figure 1). Glucose and triglyceride levels were additionally measured at the end of month 3. Venous blood was obtained from the antecubital vein between 8 and 9 a.m. after the patient had been fasting for at least 12 h. The experiments were performed in duplicate to ensure accurate results. The person performing the assays was blinded to all clinical and diagnostic information. Plasma levels of glucose, lipids, uric acid, and albumin were measured using the multi-analyzer COBAS Integra 400 Plus (Roche Diagnostics, Basel, Switzerland). Concentrations of insulin, 25-hydroxyvitamin D, total testosterone, and homocysteine were assayed by direct chemiluminescence using acridinium ester technology (ADVIA Centaur XP Immunoassay System, Siemens Healthcare Diagnostics, Munich, Germany). Levels of high-sensitivity C-reactive protein (hsCRP) were measured using an immunoassay with chemiluminescent detection (Immulite 2000XPi, Siemens Healthcare, Warsaw, Poland), while concentrations of fibrinogen were assayed by the Clauss method using an automated BCS XP analyzer (Siemens Healthcare, Warsaw, Poland). The homeostatic model assessment 1 of insulin resistance (HOMA1-IR) was computed by multiplying fasting plasma glucose (in mmol/L) by fasting insulin (in mU/L) and dividing by 22.5. The triglyceride-glucose (TyG) index was obtained through the formula: ln [triglyceride (mg/dL) × fasting plasma glucose (mg/dL)/2]. The cumulative TyG index was calculated as the weighted sum (value × time) of the mean TyG index for each 3-month interval. The urinary albumin-to-creatinine ratio (UACR) was calculated by dividing the urinary albumin concentrations by the urinary creatinine concentrations and expressed in mg/g. The 10-year cardiovascular risk was calculated using the Framingham Risk Score (FRS).

### 2.4. Statistical Analysis

All continuous variables were log-transformed before analysis to normalize skewed distributions. Between-group comparisons were carried out using analysis of variance followed by Bonferroni post hoc tests. The means and standard deviations within the same group were compared using paired *t*-tests. Qualitative data were compared using the chi-square test. The strength of relationships between the outcome measures was estimated using Pearson’s r tests. A probability value (*p*) below 0.05 was considered significant.

## 3. Results

Sixty-five men (90%), twenty-two in group I, twenty-two in group II, and twenty-one in group III, completed the study and were statistically analyzed. A post hoc power calculation showed that the study had sufficient power (0.82). Three patients (one from each group) were withdrawn because of adverse effects associated with testosterone therapy (injection side reactions, nausea, polycythemia, and edemas). Two patients, both from group III, prematurely terminated the study because of starting treatment with an angiotensin-converting enzyme inhibitor and metformin. One patient (from group I) was withdrawn owing to changing the place of residence. The last patient, allocated to group II, was withdrawn because of non-compliance with vitamin D supplementation. All analyzed patients did not experience any adverse effects and adhered to the treatment recommendations. The average daily dose of exogenous calciferol and the average duration of vitamin D supplementation in group II were 73.8 ± 14.1 µg (2952 ± 564 U) and 43 ± 11 weeks, respectively.

At entry, there were no differences between the study group in age, smoking habits, body mass index, waist circumference, blood pressure (both systolic and diastolic), TyG index, and daily calciferol intake with food (Table 1). Group I differed from the remaining groups in baseline values of 25-hydroxyvitamin D, hsCRP, fibrinogen, homocysteine, UACR, and FRS, but not in total testosterone, glucose, HOMA1-IR, total cholesterol, HDL cholesterol, LDL cholesterol, triglycerides, and uric acid. There were no differences in these parameters between groups II and III (Table 2).

In all study groups, testosterone increased total testosterone levels and did not affect 25-hydroxyvitamin D levels. In group I, testosterone reduced HDL cholesterol and hsCRP but did not affect the remaining assayed variables. In groups II and III, the drug reduced HOMA1-IR, LDL cholesterol, uric acid, hsCRP, homocysteine, fibrinogen, UACR, and FRS. In both groups, testosterone produced a neutral effect on glucose, total cholesterol, and triglycerides (Table 2). There were differences between group I and the remaining groups in the percentage changes in HOMA1-IR, total cholesterol, HDL cholesterol, LDL cholesterol, uric acid, hsCRP, homocysteine, fibrinogen, UACR, and FRS (Table 3). Testosterone did not affect smoking habits, body mass index, waist circumference, and blood pressure. Group I differed from groups II and III in the follow-up values of 25-hydroxyvitamin D, HOMA1-IR, HDL cholesterol, LDL cholesterol, uric acid, hsCRP, homocysteine, fibrinogen, UACR, and FRS (Table 2). The cumulative TyG index was higher in group I (44.15 ± 2.91) than in the remaining groups (42.27 ± 2.56 in group II and 42.38 ± 2.46 in group III).

At baseline, the TyG index, HOMA1-IR, LDL cholesterol, uric acid, hsCRP, homocysteine, fibrinogen, UACR, and FRS inversely correlated with total testosterone levels (r values between −0.301 [*p* = 0.0488] and −0.404 [*p* = 0.0015] in group I, between −0.341 [*p* = 0.0285] and −0.451 [*p* = 0.0004] in group II, and between −0.352 [*p* = 0.0254] and −0.472 [*p* = 0.0002] in group III) and with 25-hydroxyvitamin D levels (r values between −0.368 [*p* = 0.0184] and −0.424 [*p* = 0.0010] in group I, between −0.314 [*p* = 0.0354] and −0.462 [*p* = 0.0003] in group II, and between −0.341 [*p* = 0.0293] and −0.451 [*p* = 0.0004] in group III). There were also positive correlations between testosterone and HDL cholesterol (r values between 0.328 [*p* = 0.0326] and 0.371 [*p* = 0.0151], depending on the group) and between 25-hydroxyvitamin D and HDL cholesterol (r values between 0.318 [*p* = 0.0344] and 0.386 [*p* = 0.0107], depending on the group). Treatment-induced changes in HOMA1-IR, LDL cholesterol, uric acid, hsCRP, homocysteine, fibrinogen, UACR, and FRS positively correlated with the increase in testosterone concentration (r values between 0.298 [*p* = 0.0496] and 0.398 [*p* = 0.0013] in group I, between 0.375 [*p* = 0.0149] and 0.483 [*p* = 0.0001] in group II, and between 0.381 [*p* = 0.0102] and 0.492 [*p* < 0.0001] in group III) and with baseline values of 25-hydroxyvitamin D (r values between 0.380 [*p* = 0.0141] and 0.451 [*p* = 0.0004] in group I, between 0.388 [*p* = 0.0095] and 0.495 [*p* < 0.0001] in group II, and between 0.390 [*p* = 0.0088] and 0.502 [*p* < 0.0001] in group III). In group I, the impact of testosterone on HDL cholesterol positively correlated with baseline 25-hydroxyvitamin D (r = 0.415 [*p* = 0.0011]). The impact of treatment on the outcome variables did not correlate with daily calciferol intake with food and in group II with vitamin D dose and supplementation duration.

## 4. Discussion

The present study provides some clinically relevant information. Firstly, testosterone substitution in men with LOH, despite its imperfections, is likely to reduce cardiometabolic risk. In patients with normal calciferol homeostasis, the drug improved insulin sensitivity, reduced LDL, decreased plasma levels of all non-lipid risk factors, and reduced urinary loss of albumin. Considering the exclusion criteria, this finding cannot be explained by the impact of other drugs with cardiometabolic properties or interacting with endogenous or exogenous testosterone. The association between these findings and the impact of testosterone is supported by the presence of positive correlations between treatment-induced changes in HOMA1-IR, LDL cholesterol, uric acid, hsCRP, homocysteine, fibrinogen, and UACR and the impact of the substitution on plasma testosterone concentration. What is more, normalization of testosterone concentration caused by intramuscular injections of this hormone was accompanied by the follow-up values of all measured parameters within normal limits. This finding supports the rationale for testosterone substitution in all men with impaired production if they do not have contraindications to this therapy.

Secondly, patients with both LOH and low vitamin D status were characterized by higher values of hsCRP, homocysteine, fibrinogen, and UACR than men with LOH and normal calciferol homeostasis. All these variables belong to important cardiometabolic risk factors [39,40,41,42], and their elevated levels suggest increased susceptibility of vitamin D-deficient subjects to cardiovascular disease, metabolic syndrome, and type 2 diabetes. Thus, this finding suggests additive effects of testosterone deficiency and impaired calciferol homeostasis on cardiometabolic risk. Two questions need to be addressed. Firstly, between-group differences were observed despite matching all groups of patients for age, BMI, and baseline testosterone concentrations. No differences in baseline BMI may partially explain why between-group differences in HOMA1-IR, which is a validated method to estimate insulin sensitivity [43], were not statistically significant, as well as why the same relationships were observed for the TyG index, assessing insulin sensitivity by combining triglyceride and fasting glucose levels [44]. Secondly, differences in hsCRP, homocysteine, fibrinogen, and UACR were observed despite the fact that patients with vitamin D insufficiency were characterized by relatively discrete abnormalities of calciferol homeostasis. This finding, as well as correlations between 25-hydroxyvitamin D levels and most of the remaining outcome measures, suggests that cardiovascular risk may be more pronounced in individuals with untreated vitamin D deficiency (25-hydroxyvitamin D below 20 ng/mL) than in vitamin D-deficient subjects, but such patients were not included in our study. Moreover, our findings suggest that from the cardiometabolic point of view, even mild disturbances in vitamin D homeostasis should not be left untreated.

The novel and most important finding of the current study is that low vitamin D status weakened the effect of testosterone substitution on cardiometabolic risk. The impact of testosterone in vitamin D-insufficient men was limited to a decrease in hsCRP, which was less expressed than in individuals with normal calciferol homeostasis and counterbalanced by a potentially negative effect on HDL cholesterol. What is more, testosterone did not induce any changes in HOMA1-IR, uric acid, homocysteine, fibrinogen, and UACR in vitamin D-insufficient subjects, though such changes were observed in men with 25-hydroxyvitamin D levels within the reference range. Vitamin D-insufficient males also had the highest cumulative TyG index, which seems to be an independent predictor of cardiometabolic risk [44]. Lastly, the included patients were characterized by the presence of negative correlations between 25-hydroxyvitamin D and most of the assessed risk factors. Thus, it seems justified to routinely assess 25-hydroxyvitamin D in all patients who are candidates for testosterone substitution. Leaving low vitamin D status without correction may markedly reduce cardiometabolic benefits associated with testosterone replacement. It is possible that differences in the proportions of individuals with calciferol insufficiency (or deficiency) may partially explain differences in the effects of this therapy on hard cardiovascular endpoints between various studies [13,14,15,16,17].

Fourthly, patients with low vitamin D status differed from the remaining groups of patients in the impact of testosterone replacement therapy on plasma lipids, which was beneficial only in patients with normal calciferol homeostasis. In vitamin D-insufficient patients, this effect was limited to a potentially unfavorable decrease in HDL cholesterol, while in men with normal calciferol homeostasis, the supplementation reduced LDL cholesterol, and this effect was not counterbalanced by changes in HDL cholesterol. Although the overall effect on plasma lipids was modest, the significant differences in the follow-up lipid profile contributed to between-group differences in the effect of testosterone on FRS, estimating the 10-year global risk of a cardiovascular event in patients between 30 and 79 years with no history of coronary heart disease or diabetes [45]. Interestingly, testosterone replacement induced a significant decrease in this score in men with normal calciferol homeostasis despite not causing significant changes in smoking and blood pressure, which are also used to calculate this parameter.

Fifthly, it deserves underlining that there were no differences in cardiometabolic risk factors between both groups of men with normal calciferol status, though one group of patients with normal 25-hydroxyvitamin D levels was characterized by vitamin D deficiency or vitamin D insufficiency in the past. Similar values were observed both at baseline and at follow-up. This interesting observation indicates that effective vitamin D supplementation may normalize cardiometabolic risk and cardiometabolic response to testosterone replacement therapy, and this effect is a consequence of restoration of normal calciferol status. It appears rather unlikely that the obtained results reflect a specific action of exogenous vitamin D or pharmacokinetic interactions between preparations of exogenous calciferol and testosterone. In line with this explanation, there were no correlations between the assessed cardiometabolic risk factors and the daily vitamin D dose and duration of calciferol treatment. Moreover, the study groups did not differ statistically in calciferol intake from food. Thus, the obtained results allow us to conclude that the unfavorable impact of low vitamin D status on testosterone action, at least at an early stage, may be fully reversible after normalization of calciferol homeostasis.

Many men considered for enrollment were characterized by currently low 25-hydroxyvitamin D levels (20%) or previous vitamin D deficiency/insufficiency that had been an impetus for calciferol supplementation (39%). Thus, it seems that low testosterone production may predispose to low vitamin D status, and this hypothesis is in line with our previous findings [37,46] and observations from other research centers [47,48]. We observed lower 25-hydroxyvitamin D concentrations in men with hypotestosteronemia than in their peers with normal testosterone levels [37] and reported that this effect was mediated by the androgen receptor [46]. In turn, Foresta et al. observed that unilateral orchidectomy in humans reduced 25-hydroxyvitamin D levels [48], while administration of testosterone raised circulating levels of 25-hydroxyvitamin D in men with bilaterally removed gonads [47]. The relationship between testosterone and calciferol may be, however, bidirectional. Vitamin D was found to increase the testosterone/LH ratio in vitamin D-deficient men [34], while calciferol administration was reported to increase testosterone synthesis in adult human testicular cell cultures [35]. In the current study, there were no statistically significant differences between the study groups in circulating levels of this hormone. At baseline, this can be easily explained by matching the study groups for testosterone concentration. A more challenging question is to explain the reasons for no between-group differences in follow-up levels of this hormone. We assume that a similar increase in testosterone levels may reflect the same dose of exogenous testosterone esters in all study groups.

We can only hypothesize about a mechanistic explanation of our findings. The beneficial effects of testosterone replacement on the investigated biomarkers are likely to belong to its genomic effects. In a pilot preceding our previous study [49], cardiometabolic effects were absent during the first two months of testosterone substitution, which is in sharp contrast with a rapid onset of non-genomic effects [11,12]. The androgen receptor was also found to mediate metabolic effects of its agonists in other human and experimental studies [50,51]. Abnormal levels of total and LDL cholesterol and impaired insulin sensitivity were present in a large subset of patients with complete androgen insensitivity syndrome, which is caused by loss-of-function mutations in the androgen receptor gene [50]. In turn, androgen receptor-knockout mice exposed to a high-fat diet were found to be characterized by the earlier development of atherosclerotic changes in the aorta, impaired insulin sensitivity, and atherogenic dyslipidemia in comparison to control mice with the intact androgen receptor gene [51]. Lastly, the androgen receptor is abundantly expressed in the liver, kidneys, and inflammatory cells, playing an important role in the production of the investigated cardiometabolic risk factors [52]. No between-group differences in the increase in testosterone levels argue against the impact of vitamin D on the absorption of exogenous testosterone, the production of the endogenous hormone (impaired due to LOH), and testosterone metabolism. The much more likely explanation for between-group differences in the outcome measures is that testosterone and vitamin D interact at the level of target tissues, which is supported by some findings. In vitro observations suggest potential signaling cross-talk between the androgen receptor and the vitamin D receptor [53]. Expression of the androgen receptor in the prostate was found to be induced by calcitriol in a dose-dependent manner [54]. The possible link between calciferol and testosterone in the regulation of mood in middle-aged and elderly men has been recently postulated by another research group [55]. Lastly, although no study determined the impact of calciferol and its metabolites on the expression of the androgen receptor in the extraprostatic tissues, the vitamin D receptor is detectable in tissues expressing the androgen receptor, including hepatocytes, kidneys, and inflammatory cells [52,56]. Although these findings suggest interconnections between the androgen and vitamin D pathways, we cannot exclude the association between our findings and the conversion of testosterone to estradiol because the active form of vitamin D exerted a stimulatory effect on aromatase activity in males [57]. Endogenous estrogens are considered protective against cardiovascular morbidity and related mortality in women [58]. It is possible that increased estradiol production may have beneficial cardiometabolic effects also in men, partially mediating the impact of androgens.

Several potential study limitations need to be acknowledged. Firstly, due to the small number of patients (though exceeding the requirements for sample size), our observations should be interpreted as hypothesis-generating and warranting large-scale validation. Secondly, assessment of only surrogate outcomes in the current study provides only probabilistic answers, but not guarantees of clinically relevant findings. Thirdly, because of the study design (non-randomized cohort study), the results might have been potentially influenced by selection and confounding bias. Fourthly, we cannot completely exclude a late-appearing effect of non-pharmacological treatment. Fifthly, excluding patients with untreated severe vitamin D deficiency makes it impossible to draw firm conclusions about testosterone action in this group of patients. Lastly, the study design does not allow us to explain molecular mechanisms mediating the inhibitory effect of low vitamin D status on testosterone action in men with low testosterone production.

## 5. Conclusions

In case of coexisting untreated vitamin D insufficiency, men with LOH were characterized by increased values of some cardiometabolic risk factors, including hsCRP, homocysteine, fibrinogen, and UACR. Low vitamin D status, unless effectively treated, attenuated the impact of testosterone substitution on most assessed outcome measures. However, testosterone effects were preserved if low vitamin D status was effectively supplemented. The replacement-induced decrease in uric acid, LDL cholesterol, hsCRP, homocysteine, fibrinogen, and UACR positively correlated with 25-hydroxyvitamin D concentrations. The obtained results suggest that even discrete abnormalities in calciferol homeostasis may impair the cardiometabolic response to testosterone replacement therapy in men with LOH. Thus, our findings shed a new light on the association between androgen and vitamin D status in middle-aged or elderly men. They should encourage performing a larger, well-designed multicenter clinical study that would overcome important limitations of the current pilot study.

## Figures and Tables

**Table 1:** Baseline characteristics of men participating in the study.

Variable	Group I	Group II	Group III
**Number (n)**	22	22	21
**Age** (years)	59 ± 8	60 ± 8	58 ± 9
**Smokers** (%)/**number of cigarettes a day** (n)**/duration of smoking** (years)	45/11 ± 7/30 ± 15	41/10 ± 6/32 ± 17	43/9 ± 7/31 ± 14
**Body mass index** (kg/m^2^)	28.8 ± 5.5	27.7 ± 5.3	27.5 ± 5.8
**Waist circumference** (cm)	102 ± 10	99 ± 10	98 ± 8
**Systolic blood pressure** (mmHg)	135 ± 18	131 ± 20	130 ± 22
**Diastolic blood pressure** (mmHg)	87 ± 8	84 ± 8	84 ± 7
**TyG index**	4.83 ± 0.17	4.82 ± 0.15	4.85 ± 0.18
Daily calciferol intake with food * (µg)	10.4 ± 5.2	9.6 ± 4.8	11.8 ± 5.8

Unless otherwise stated, the data are shown as the mean ± standard deviation. Group I: vitamin D-naive individuals with 25-hydroxyvitamin D levels between 20 and 30 ng/mL; group II: males with 25-hydroxyvitamin D levels between 30 and 60 ng/mL receiving vitamin D supplementation because of previous low vitamin D status; group III: vitamin D-naïve subjects with 25-hydroxyvitamin D levels between 30 and 60 ng/mL. * Not counting vitamin D contained in tablets.

**Table 2:** The effect of testosterone replacement on the investigated variables in men with LOH and different vitamin D status.

Variable	Group I	Group II	Group III
**Total testosterone** (nmol/L)			
*Baseline*	8.3 ± 1.5	7.8 ± 1.9	8.1 ± 1.7
*Follow-up*	16.2 ± 3.1 ^#^	15.9 ± 2.9 ^#^	16.9 ± 3.5 ^#^
**25-hydroxyvitamin D** (ng/mL)			
*Baseline*	24.8 ± 2.5 *	46.0 ± 7.3	45.2 ± 8.0
*Follow-up*	25.2 ± 2.8 *	46.8 ± 6.9	44.9 ± 7.5
**Glucose** (mg/dL)			
*Baseline*	94 ± 12	92 ± 12	91 ± 11
*Follow-up*	92 ± 11	88 ± 12	87 ± 12
**HOMA1-IR**			
*Baseline*	3.4 ± 1.2	3.2 ± 0.9	3.0 ± 0.9
*Follow-up*	3.2 ± 1.0 *	2.1 ± 0.8 ^#^	1.9 ± 0.9 ^#^
**Total cholesterol** (mg/dL)			
*Baseline*	205 ± 34	208 ± 35	213 ± 40
*Follow-up*	202 ± 30	193 ± 32	196 ± 29
**HDL cholesterol** (mg/dL)			
*Baseline*	48 ± 9	49 ± 9	51 ± 11
*Follow-up*	40 ± 10 *^, #^	50 ± 11	51 ± 12
**LDL cholesterol** (mg/dL)			
*Baseline*	121 ± 27	122 ± 25	125 ± 21
*Follow-up*	124 ± 23 *	108 ± 22 ^#^	109 ± 24 ^#^
**Triglycerides** (mg/dL)			
*Baseline*	168 ± 61	174 ± 70	180 ± 67
*Follow-up*	179 ± 71	172 ± 64	175 ± 75
**Uric acid** (mg/dL)			
*Baseline*	4.9 ± 1.7	4.7 ± 1.9	5.2 ± 2.0
*Follow-up*	4.4 ± 1.6 *	3.4 ± 1.6 ^#^	3.4 ± 1.4 ^#^
**hsCRP** (mg/L)			
*Baseline*	3.9 ± 1.2 *	3.2 ± 1.0	3.0 ± 1.1
*Follow-up*	3.2 ± 0.9 *^, #^	1.3 ± 0.8 ^#^	1.4 ± 0.7 ^#^
**Homocysteine** (μmol/L)			
*Baseline*	34.1 ± 11.9 *	23.6 ± 9.5	22.4 ± 8.8
*Follow-up*	31.8 ± 10.2 *	12.8 ± 7.5 ^#^	11.7 ± 8.4 ^#^
**Fibrinogen** (mg/dL)			
*Baseline*	438 ± 85 *	370 ± 78	358 ± 91
*Follow-up*	420 ±95 *	295 ± 101 ^#^	283 ± 79 ^#^
**UACR** (mg/g)			
*Baseline*	32.8 ± 12.5 *	22.8 ± 7.6	23.5 ± 8.0
*Follow-up*	31.2 ± 10.9 *	11.5 ± 7.1 ^#^	12.8 ± 6.9 ^#^
**FRS** (%)			
*Baseline*	13.7 ± 2.8 *	12.2 ± 2.9	11.9 ± 3.2
*Follow-up*	14.2 ± 3.5 *	10.5 ± 3.4 ^#^	10.0 ± 3.0 ^#^

The data are shown as the mean ± standard deviation. Group I: vitamin D-naive individuals with 25-hydroxyvitamin D levels between 20 and 30 ng/mL; group II: males with 25-hydroxyvitamin D levels between 30 and 60 ng/mL receiving vitamin D supplementation because of previous low vitamin D status; group III: vitamin D-naïve subjects with 25-hydroxyvitamin D levels between 30 and 60 ng/mL. * *p* < 0.05 vs. values at the same time point in groups II and III; ^#^ *p* < 0.05 vs. baseline values in the same study group.

**Table 3:** Percentage changes from baseline in the investigated variables in men with LOH and different vitamin D status.

Variable	Group I	Group II	Group III
**Δ Total testosterone**	95 ± 35	104 ± 40	109 ± 46
**Δ 25-hydroxyvitamin D**	2 ± 8	2 ± 7	−1 ± 5
**Δ Glucose**	−2 ± 6	−4 ± 7	−4 ± 6
**Δ HOMA1-IR**	−6 ± 18 *	−34 ± 18	−37 ± 16
**Δ Total cholesterol**	−1 ± 8 *	−7 ± 10	−8 ± 11
**Δ HDL cholesterol**	−20 ± 9 *	2 ± 8	0 ± 12
**Δ LDL cholesterol**	2 ± 9 *	−11 ± 10	−13 ± 12
**Δ Triglycerides**	7 ± 20	−1 ± 19	−3 ± 24
**Δ Uric acid**	−10 ± 18 *	−28 ± 18	−35 ± 24
**Δ hsCRP**	−18 ± 21 *	−59 ± 30	−53 ± 28
**Δ Homocysteine**	−7 ± 20 *	−46 ± 25	−48 ± 19
**Δ Fibrinogen**	−4 ± 16 *	−20 ± 20	−21 ± 18
**Δ UACR**	−5 ± 15 *	−50 ± 23	−46 ± 24
**Δ FRS**	4 ± 12 *	−14 ± 16	−16 ± 15

The data are shown as the mean ± standard deviation. Group I: vitamin D-naive individuals with 25-hydroxyvitamin D levels between 20 and 30 ng/mL; group II: males with 25-hydroxyvitamin D levels between 30 and 60 ng/mL receiving vitamin D supplementation because of previous low vitamin D status; group III: vitamin D-naïve subjects with 25-hydroxyvitamin D levels between 30 and 60 ng/mL. * *p* < 0.05 vs. percentage changes in groups II and III.

## Data Availability

The data that support the findings of this study are available from the corresponding author upon reasonable request. They are not publicly available due to privacy and legal restrictions.
